# The impact of acute diarrhea on the coagulation status of patients with vitamin K antagonists

**DOI:** 10.1038/s41598-021-91316-x

**Published:** 2021-06-03

**Authors:** Johannes Schweinfurth, Alexander Bauer, Frederic Bauer, Felix Sebastian Seibert, Benjamin Rohn, Maximilian Seidel, Sebastian Bertram, Ulrik Stervbo, Nina Babel, Timm Henning Westhoff

**Affiliations:** Medical Department I, Marien Hospital Herne, University Hospital of the Ruhr-University Bochum, Hölkeskampring 40, 44625 Herne, Germany

**Keywords:** Risk factors, Drug regulation

## Abstract

Acute diarrhea is associated with a reduced absorption of both vitamin K antagonists (VKA) and vitamin K itself. To date, the net effect on the coagulation status of subjects with VKA remains elusive. We performed a systematic retrospective single-center analysis using an electronic data extraction approach to identify subjects with plasmatic anticoagulation (either VKA or direct oral anticoagulant (DOAC)) and diarrhea in a German University Hospital over a period of eight years. Acute diarrhea and complete documentation of coagulation status on admission were defined as inclusion criteria, anticoagulation other than VKA/DOAC and obvious inadherence as exclusion criteria. Subjects with VKA/DOAC admitted for hypertension served as control group. Data extraction yielded 356 subjects with gastrointestinal diagnoses and 198 hypertensive subjects, 55 and 83 of whom fulfilled all in- and exclusion criteria. INR values of subjects with VKA were significantly higher in subjects with diarrhea than in hypertensive controls (4.3 ± 3.7 vs. 2.3 ± 0.7, *p* < 0.001). The distribution of subjects having INR values lower, higher or within the target range differed significantly among groups with a substantially higher prevalence of overanticoagulation in the diarrhea group (46.4% vs. 14.3%, *p* < 0.001). In a multinomial logistic regression model, acute diarrhea was significantly associated with overanticoagulation (odds ratio 7.2, 95% confidence interval 2.163–23.921; *p* < 0.001), whereas age, sex, creatinine, and indication of anticoagulation were not (*p* > 0.05 each). Acute diarrhea is associated with a highly increased risk for overanticoagulation in patients with VKA. Thus, gastroenteritis necessitates a close monitoring of INR in order to identify subjects needing a temporary pause of VKA therapy.

## Introduction

Phenprocoumon and warfarin exert their anticoagulatory effects by inhibiting the vitamin K dependent synthesis of clotting factors in the liver. There are numerous factors that may alter the resorption, metabolism and response to Vitamin K antagonists (VKA). Thus, liver disease, hyperthyroidism, and chronic kidney disease or acute states like decompensated heart failure and fever can increase the response to VKA^1^. Moreover, several drug interaction have to be taken into account. E. g., antibiotics like chinolons and contrimoxazole are associated with an increased risk of haemorrhage^2^.

Although these drugs have been standard of care for decades, little is known about the effects of acute episodes of diarrhea on the coagulation status of subjects with VKA. On the one hand diarrhea leads to decreased absorption of the drug, potentially resulting in ineffective anticoagulation. On the other hand, diarrhea reduces the absorption of vitamin K itself, which could lead to temporary overanticoagulation. It remains elusive, which effect overweighs the other.

So far, only isolated case reports are available on this topic. In 1994, a physician reported about himself taking warfarin and suffering from overanticoagulation with excessively increased international normalized ratio (INR) values during an episode of giardiasis^3^. Over the following 20 years four further case reports indicated that diarrhea may lead to overanticoagulation in subjects taking VKA^4–7^. Despite the high clinical relevance of this issue, there is neither a case series nor a systemic retrospective analysis on this issue so far. The present work constitutes the first systematic analysis on the effects of new-onset diarrhea on coagulation parameters in patients with VKA.

## Methods

### Design and protocol

Subjects with oral anticoagulation that were admitted to the Medical Department of a German University Hospital from 2011–2018 for acute gastroenteritis were enrolled in this retrospective analysis. Subjects admitted for hypertension served as control group.

Data extraction was performed electronically in an anonymized manner based on the diagnosis coded at discharge and submitted to the health insurance. The study was approved by the ethics committee of the Ruhr-University Bochum (20-6963-BR) and informed consent was waived by the ethics committee of the Ruhr-University Bochum. The protocol was in accordance with institutional and national guidelines. All subjects with a diagnosis related group (DRG) code "G67A-C" between 2011 and 2018 were identified. These DRGs comprise e. g. acute gastroenteritis, esophagitis, gastrointestinal bleeding, diverticulitis, ulcera ventriculi and duodeni. Those ones with coincident documentation of the code for plasmatic anticoagulation (Z92.1) were enrolled in the analysis. In a second step, those ones with acute diarrhea were identified by the patient’s chart (gastroenteritis group). The other subjects were excluded from further analysis. Finally, it was checked, whether the subject was administered a VKA, a direct oral anticoagulant (DOAC), low molecular heparin or other anticoagulants until admission to the hospital. Acute diarrhea was defined as mandatory inclusion criterion, obvious inadherence (either documented in patient’s history or INR < 1.3 despite VKA), anticoagulation other than VKA/DOAC, and lack of INR data at admission were defined as exclusion criterion. Subjects admitted to hospital due to a hypertensive episode (I10.01/I10.11) receiving plasmatic anticoagulation served as control population. Overanticoagulation was defined as an INR > 3.5 for subjects with mechanical heart valves and > 3.0 for atrial fibrillation, venous thrombosis, and pulmonary embolism.

### Statistical analysis

Numeric data are presented as mean ± standard deviation. Data were tested for normal distribution by the Kolmogorov–Smirnov test. Intergroup differences (gastroenteritis with VKA, gastroenteritis with DOAC, control with VKA, control with DOAC) in numeric normally distributed data were analyzed by ANOVA. Categorical data were compared by Pearson Chi squared test. Multinomial logistic regression analysis with “overanticoagulation” as dependent variable was performed for patients with VKA using creatinine, age, C-reactive protein (CRP), diarrhea, indication for anticoagulation, and sex as covariates/factors. The distribution of subjects with over-, under-, and normoanticoagulation in the gastroenteritis vs. control group was compared by Pearson Chi squared test. INR values among VKA patients with diarrhea vs. control were compared using unpaired two-tailed Student's *t* test. All statistical analyses were done using SPSS Statistics 25 (SPSS Inc, Chicago, Illinois, USA) and Prism 5 (GraphPad Software, La Jolla, California, USA). *p* < 0.05 was regarded statistically significant.

## Results

**Figure 1:**
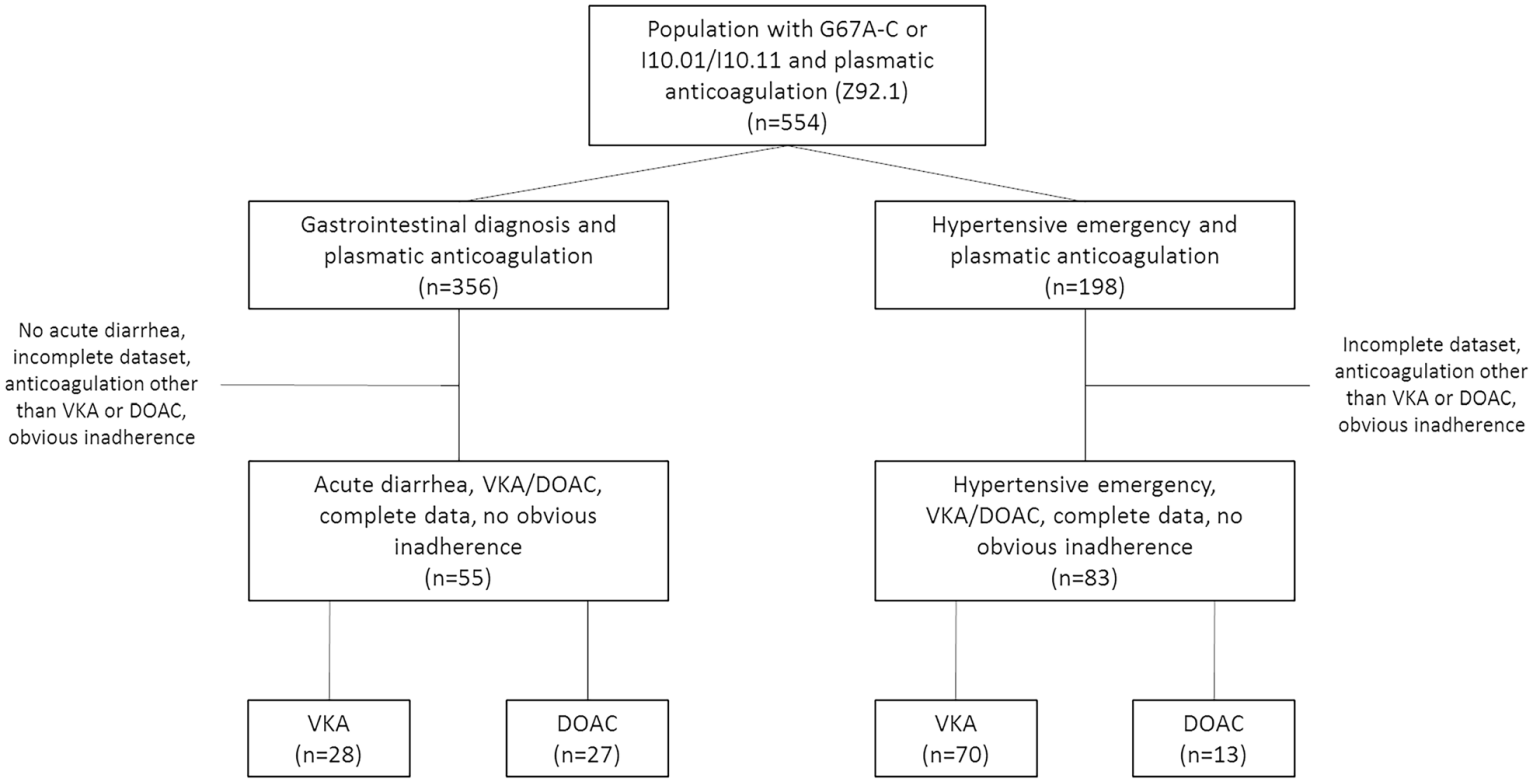
Study population.

Electronic data extraction yielded an overall population of 554 subjects with the code for anticoagulation, who were admitted to hospital for either a bowel-related diagnosis (G67A-C, n = 356) or hypertension (I10.01/I10.11, n = 198). 83 of those ones with the primary diagnosis of hypertension fulfilled in- and exclusion criteria and served as control group. 70 (84.3%) were administered phenprocoumon, 13 (15.7%) received a DOAC. Among those with a bowel-related diagnosis, 55 suffered from acute diarrhea, had INR data on admission, anticoagulation with either VKA or DOAC and no obvious inadherence. These subjects constituted the gastroenteritis group. 28 (50.9%) were administered a VKA, 27 (49.1%) received a DOAC. A flow sheet of the composition of the study population is provided in Fig. 1.

Mean age of the overall study population was 77.0 ± 10.7 years. Age differed significantly between the four groups (gastroenteritis with VKA, gastroenteritis with DOAC, control with VKA, control with DOAC; *p* 0.04). Subjects with DOACs were older than those ones with VKA. Atrial fibrillation was the most frequent indication for anticoagulation (81.9%). 7.2% of the patients had anticoagulation due to prosthetic valves, the rest for either venous thrombosis or pulmonary embolism. There was no intergroup difference in the indications of anticoagulation. Moreover, gastroenteritis and control group were homogenous for sex and white blood cell count (*p* > 0.05 each). Gastroenteritis was associated with higher CRP and creatinine values (*p* < 0.05 each). Noteworthy, none of the patients with VKA and diarrhea had antibiotics at the time of laboratory examinations (admission). Table 1 presents the epidemiological data and the coagulation status of the study population. Norovirus, rotavirus, Campylobacter jejuni, enteropathogenic Escherichia coli (EPEC), and clostridioides were identified as infectious agents.

**Table 1:** Characterization of study population. Subjects with gastroenteritis versus subjects with hypertension serving as control group.

Parameter	Overall study population (n = 138)	Gastroenteritis		Control		*p*
		VKA (n = 28)	DOAC (n = 27)	VKA (n = 70)	DOAC (n = 13)	
Female	95 (68.9%)	15 (53.6%)	19 (70.4%)	49 (70.0%)	12 (92.3%)	0.09
Male	43 (31.1%)	13 (46.4%)	8 (29.6%)	21 (30.0%)	1 (7.7%)	
Age (years)	77.0 ± 10.7	76.8 ± 11.3	81.7 ± 10.7	75.0 ± 10.1	77.0 ± 10.7	0.04
**Indication of anticoagulation**						
Atrial fibrillation	113 (81.9%)	21 (75.0%)	21 (77.8%)	59 (84.3%)	12 (92.3%)	0.75
Venous thrombosis	11 (8.0%)	2 (25.0%)	3 (11.1%)	5 (7.1%)	1 (7.7%)	
Pulmonary embolism	4 (2.9)	2 (25.0%)	0 (0.0%)	2 (2.9%)	0 (0.0%)	
Valve	10 (7.2%)	3 (10.7%)	3 (11.1%)	4 (5.7%)	0 (0.0%)	
**Laboratory data**						
Serum creatinine (mg/dl)	1.3 ± 0.6	1.6 ± 0.9	1.3 ± 0.6	1.2 ± 1.1	1.1 ± 0.32	0.02
CRP (mg/dl)	2.3 ± 5.1	4.3 ± 7.7	5.2 ± 7.0	0.7 ± 1.2	0.8 ± 1.0	< 0.001
White blood count (/nl)	10.2 ± 12.8	9.5 ± 5.7	9.1 ± 5.7	11.4 ± 17.3	7.9 ± 1.7	0.74
INR on admission	2.5 ± 2.0	4.3 ± 3.7	1.5 ± 0.6	2.3 ± 0.7	1.3 ± 0.6	< 0.001
Quick value on admission (PT, %)	42.9 ± 23.8	24.4 ± 12.9	62.6 ± 21.8	35.9 ± 14.2	79.5 ± 22.2	< 0.001
Partial Thromboplastin Time on admission (PTT, sec)	40.0 ± 13.6	42.9 ± 10.5	31.3 ± 6.6	42.7 ± 13.8	37.2 ± 20.5	0.001
INR at discharge	2.4 ± 3.6	3.6 ± 7.8	2.5 ± 1.9	2.0 ± 0.7	1.0 ± 0.5	0.26

Numeric data are presented as mean and standard deviation. Numeric data of the four groups were tested for statistically significant differences by ANOVA. Categorical data were compared by Pearson Chi squared test. *p* < 0.05 was regarded statistically significant.

*VKA* vitamin K antagonist; *DOAC* direct-acting oral anticoagulants (DOAC).

**Figure 2:**
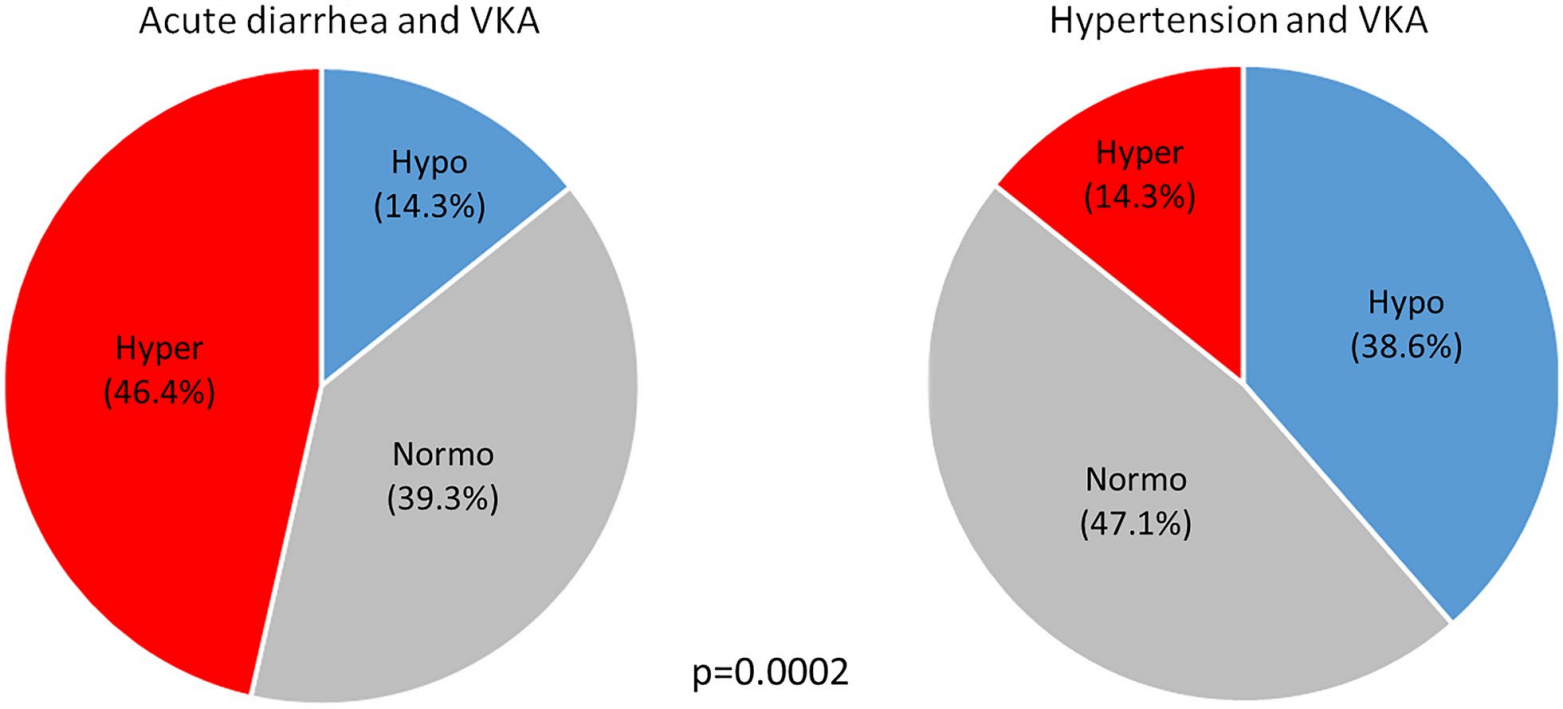
Proportion of subjects with vitamin K antagonists (VKA) having an INR < therapeutic range (Hypo), an INR > therapeutic range (Hyper), and within the therapeutic range (Normo) among those with acute diarrhea and the hypertensive control group. Intergroup differences in the distribution of the three categories were analyzed by Pearson Chi squared test. *P* < 0.05 was regarded significant.

**Figure 3:**
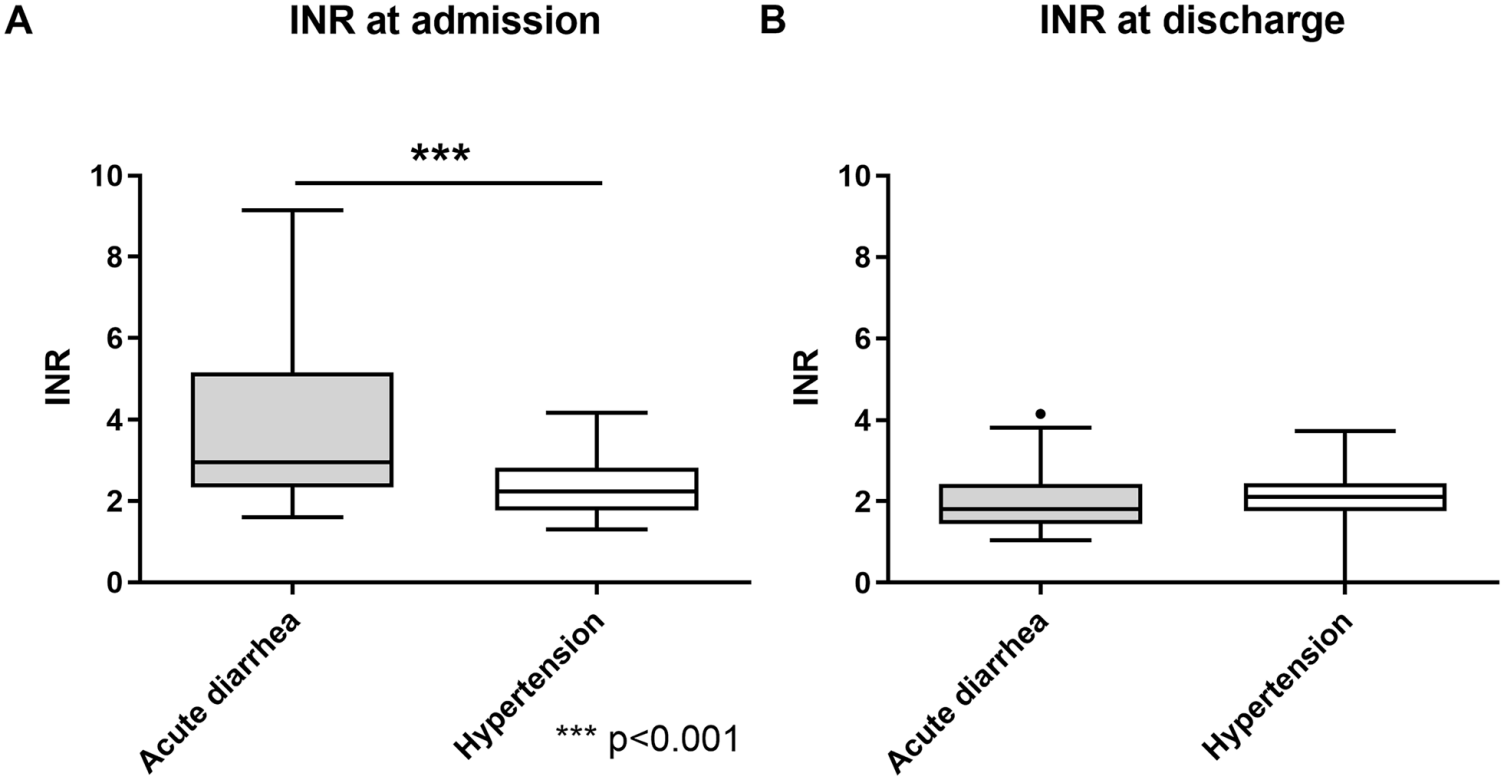
Individual international normalized ratio (INR) values (**A**) at admission and (**B**) at discharge in subjects with acute diarrhea and hypertension (control group) receiving vitamin K antagonists. ***p < 0.001 (unpaired two-tailed *t* test).

The prevalence of overanticoagulation at admission was 46.4% in the gastroenteritis and 14.3% in the control group. Figure 2 shows the distribution of subjects with hyper-, hypo-, and normoanticoagulation in these two groups, which differed significantly (*p* < 0.001). As illustrated by Fig. 3, subjects with VKA had significantly higher INR values in the gastroenteritis than in the control group (4.3 ± 3.7 vs. 2.3 ± 0.7, *p* < 0.001). Accordingly, Quick values were lower on admission in VKA subjects with gastroenteritis than in hypertensive controls (24.4 ± 12.9% vs. 35.9 ± 14.2%, *p* < 0.001). At discharge from hospital, INR values did not differ anymore (*p* 0.26).

A multinomial logistic regression was used to examine the association of serum creatinine, age, CRP, diarrhea, indication for anticoagulation, and sex on the status of overanticoagulation in patients with VKA. As shown in Table 2, acute diarrhea was highly significantly associated with overanticoagulation (Odds ratio 7.2, 95% confidence interval 2.163–23.921; *p* 0.001). None of the other parameters was associated with an INR beyond the target range.

**Table 2:** Multinomial logistic regression analysis on patients with VKA using overanticoagulation as dependent binary variable.

Parameter	Odds ratio (Exp(B))	95% Confidence interval	*p*
Creatinine	0.986	0.439–2.218	0.974
C-reactive protein	1.134	0.932–1.380	0.208
Age	1.021	0.967–1.078	0.451
Diarrhea	7.193	2.163–23.921	0.001
Sex	1.084	0.339–3.464	0.892
*Indication for anticoagulation* Atrial fibrillation	1.126	0.113–11.251	0.920
Deep vein thrombosis/pulmonary embolism	0.968	0.061–15.416	0.981
Mechanical heart valve	0.813	0.033–20.088	0.899

Creatinine, CRP and age were used as covariates, diarrhea, sex and indication for anticoagulation as factors. *p* < 0.05 was regarded statistically significant.

## Discussion

The present work constitutes the first systematic analysis of the impact of acute diarrhea on the coagulation status of subjects with VKA. In line with the few available previous case reports it shows that acute episodes of diarrhea are associated with an increased risk of overanticoagulation. Subjects with VKA and diarrhea had a substantially higher risk of an INR beyond the target range than subjects with VKA that were admitted to hospital for hypertension. Acute diarrhea was associated with overanticoagulation in almost 50% of patients. Consistently, the rate of subjects with INR in the target range was lower than in the control group. These findings are of clinical relevance, since an INR excess is associated with a substantially increased bleeding risk^8–10^.

This insight is of especial importance for subjects receiving anticoagulation for mechanical heart valves. First, since the intensity of anticoagulation is higher than in subjects with atrial fibrillation or venous thrombosis, usually with an INR target range of 2.5–3.5 vs. 2.0–3.0. Thus, the risk of overanticoagulation is higher than in subjects with a lower INR target. Second, even short states of dysanticoagulation are associated with a relevant thrombembolic risk in this indication. Third, vitamin K antagonists are still the only available way of oral anticoagulation in these subjects. After the REALLIGN trial has demonstrated an increased risk of both thromboembolic events and bleeding complications for the direct thrombin inhibitor dabigatran compared to warfarin, it is unlikely that manufacturers of other DOACs will initiate another trial in this population^11^. Hence, VKA will remain standard of care in subjects with mechanical heart valves for the time being.

In the logistic regression model, the odds ratio for overanticoagulation in acute diarrhea was 7.2. Interestingly, neither age nor kidney function predicted overanticoagulation in this model. The clinical consequence of these findings is that episodes of diarrhea necessitate an intense monitoring of the coagulation state. Subjects who are trained in self-monitoring should be instructed to perform daily INR controls. Subjects without the opportunity of self-controls should be encouraged to attend their physician for extraordinary checks of INR.

Phenprocoumon is completely absorbed from the gastrointestinal tract. It circulates bound to plasma albumin and is metabolized in the liver^12^. Diarrhea is usually associated with a reduction of a drug's absorption resulting in lower plasma levels. There are few exceptions from this rule, e.g. for drugs that are metabolized in the intestine. Thus, the immunosuppressive drug tacrolimus is absorbed in the duodenum and jejunum and partially metabolized by cytochrome p450 in the intestinal mucosa. Diarrhea reduces metabolization of the drug, which apparently outweighs a potential decrease in absorption yielding increased plasma concentrations^13^. In the case of phenprocoumon and warfarin, however, there is no intestinal inactivation of the drug. It has therefore to be hypothesized that diarrhea has a stronger effect on the absorption of vitamin K than on the absorption of the drug. Indeed, diarrhea leads to a substantial reduction of vitamin K absorption^14^.

In spite of its high clinical relevance, the present study constitutes the first systematic examination on the effects of acute diarrhea on the coagulation state in subjects with vitamin K antagonists. The patent on phenprocoumon by Hoffmann–La Roche was granted more than 60 years ago in 1955. The first description of a potential risk of overanticoagulation during diarrhea occurred not until 1994. Interestingly, this case description—a physician that reported on himself during an episode of gastroenteritis—was regarded as highly relevant and was therefore published in the Lancet^3^. Nevertheless, this report did not initiate any initiatives to investigate this issue systematically and it is unlikely that there will be a prospective evaluation in the future. The present data are therefore the best available evidence to establish awareness in physicians and patients for the need of an intense laboratory monitoring of the coagulation state during episodes of diarrhea. In line with our results, three studies on various factors predicting an increased response to VKA identified diarrhea as one of them as well^15–17^. Interestingly, these studies were published from 1998 to 2008. More recent data is scarce, showing that this issue has not been investigated in more detail despite its high clinical relevance.

Our study is limited by its monocentric view and its sample size. Furthermore, the study only enrolled hospitalized patients, which likely have more intense episodes of diarrhea than outpatients. Thus, the findings might overestimate the effects on INR in outpatients. Moreover, the data extraction approach did not allow to define all the lifestyle features, drug interactions, disease states or dosing errors that might have an effect on VKA response. Finally, we only focused on acute diarrhea. We therefore cannot draw any conclusions on states of chronic diarrhea, e.g. in chronic malabsorptive diarrhea. On the other hand, the study systematically covers a range of 8 years in a Medical Department of a University Hospital and the small study size is the consequence of the strict in- and exclusion criteria. Moreover, the study design provided a control group with a very similar pattern of indications for anticoagulation and comorbidities that was admitted for other reasons. This control group minimized potential confounders factors as far as possible and allowed a quantification of the effect of diarrhea on the risk of overanticoagulation.

## Conclusion

The findings presented here will increase awareness for the high risk of INR excess in subjects with VKA and acute diarrhea. They should encourage physicians to closely monitor INR in these patients in order to identify the need for a temporary pause of VKA therapy. Moreover, physicians should encourage patients, who perform INR self-monitoring, to intensify the monitoring during acute diarrhea, e.g. in a daily manner. This approach will likely reduce the rate of adverse bleeding events.

## Data Availability

The datasets used and/or analyzed during the current study are available from the corresponding author on reasonable request.

## Abbreviations

VKA: Vitamin K antagonists
INR: International normalized ratio
DOAC: Direct oral anticoagulant
CRP: C-reactive protein
